# A CATT Negative Result after Treatment for Human African Trypanosomiasis Is No Indication for Cure

**DOI:** 10.1371/journal.pntd.0000590

**Published:** 2010-01-26

**Authors:** Veerle Lejon, Dieudonné Mumba Ngoyi, Marleen Boelaert, Philippe Büscher

**Affiliations:** 1 Department of Parasitology, Institute of Tropical Medicine, Antwerp, Belgium; 2 Institut National de Recherche Biomédicale, Kinshasa, Democratic Republic of the Congo; 3 Department of Public Health, Institute of Tropical Medicine, Antwerp, Belgium; New York University School of Medicine, United States of America

## Abstract

**Background:**

Cure after treatment for human African trypanosomiasis (HAT) is assessed by examination of the cerebrospinal fluid every 6 months, for a total period of 2 years. So far, no markers for cure or treatment failure have been identified in blood. Trypanosome-specific antibodies are detectable in blood by the Card Agglutination Test for Trypanosomiasis (CATT). We studied the value of a normalising, negative post-treatment CATT result in treated *Trypanosoma brucei (T.b.) gambiense* sleeping sickness patients as a marker of cure.

**Methodology/Principal Findings:**

The CATT/*T.b. gambiense* was performed on serum of a cohort of 360 *T.b. gambiense* patients, consisting of 242 primary and 118 retreatment cases. The CATT results during 2 years of post-treatment follow-up were studied in function of cure or treatment failure. At inclusion, sensitivity of CATT was 98% (234/238) in primary cases and only 78% (91/117) in retreatment cases. After treatment, the CATT titre decreased both in cured patients and in patients experiencing treatment failure.

**Conclusions/Significance:**

Though CATT is a good test to detect HAT in primary cases, a normalising or negative CATT result after treatment for HAT does not indicate cure, therefore CATT cannot be used to monitor treatment outcome.

## Author Summary

The 2 year follow-up period required after treatment of human African trypanosomiasis (HAT) patients is a major challenge for patients and control programmes alike. The patient should return every 6 months for lumbar puncture and cerebrospinal fluid examination since, so far, no markers for cure have been identified in blood. The Card Agglutination Test for Trypanosomiasis (CATT) is a simple, rapid test for trypanosome-specific antibody detection in blood that is extensively used in endemic areas to screen for HAT. We examined the value of a normalising CATT as a marker for treatment outcome. We observed that CATT titres decreased after treatment both in patients who experienced treatment failure as well as in cured patients. We conclude that CATT, though a good screening test, is unreliable for monitoring treatment outcome. We also showed that the sensitivity of CATT in relapse cases was as low as 78%, and as a consequence some relapse cases might be missed in screening programs if they have no clinical signs yet.

## Introduction

Since none of the drugs for human African trypanosomiasis (HAT) is 100% efficacious, it is recommended to follow-up sleeping sickness patients every 6 months after treatment, for a period of 2 years. Parasites may be difficult to detect in blood of HAT patients experiencing treatment failure, therefore assessment at follow-up visits relies mainly on lumbar puncture and examination of the cerebrospinal fluid (CSF) for presence of trypanosomes and white blood cell count. A patient is declared cured when, within 2 years, no trypanosomes have been detected and the CSF white blood cell count returned to normal [1]. Complete follow-up is seldom achieved because, when patients feel well, they are reluctant to comply to the follow-up examinations [2]–[5]. So far, no markers for cure or treatment failure after HAT treatment have been identified in blood.

The card agglutination test for trypanosomiasis (CATT) is a fast and simple agglutination test for detection of trypanosome specific antibodies in blood of *Trypanosoma brucei* (*T.b*.) *gambiense* infected patients [6]. With sensitivities between 87 and 98% and specificities of around 95%, the CATT test is extensively used in almost all HAT endemic areas for population screening, and has contributed to the current success of HAT control programs [7],[8]. Given the fact that drugs for HAT are toxic, and the specificity of CATT is limited, a confirmation step by parasitological techniques is needed [7]. Trypanosome specific antibodies, detectable by CATT have been demonstrated even 24 months after successful treatment in no less than 47% of *gambiense* HAT patients [3],[9],[10]. A positive post-treatment CATT result is therefore not indicative of treatment failure, but the predictive value of a negative CATT after treatment has hitherto not been evaluated. We explored the hypothesis that a normalising, negative post-treatment CATT result indicates cure in *gambiense* HAT and rules out treatment failure. If such CATT-normalising patients could be released from further follow-up, this would lead to major clinical and public health benefits as less lumbar punctures would be required and less patients should be followed for up to 24 months.

We report here on the pre- and post-treatment CATT serum results in a cohort of primary and retreatment HAT cases infected with *T.b. gambiense*.

## Methods

### Ethics statement

Sleeping sickness patients originate from a prospective observational study (THARSAT) [11]. The Commission for Medical Ethics of the Prince Leopold Institute of Tropical Medicine, Antwerp, Belgium and the Ethical Commission of the Ministry of Public Health, Democratic Republic of the Congo approved the study. Written informed consent was given by all study participants prior to enrolment.

### Patients

The cohort consisted of 242 primary HAT cases that had never been treated for HAT and of 118 retreatment cases previously treated for HAT, but with trypanosomes detected in CSF at inclusion. All cases were parasitologically confirmed before enrolment and were (re)treated according to the national guidelines: primary cases in first stage (n = 41) were treated with pentamidine, primary cases in second stage were treated with melarsoprol (n = 192) or eflornithine (n = 9). Retreatment cases were treated with melarsoprol (n = 7), eflornithine (n = 52), melarsoprol nifurtimox combination therapy (n = 57), melarsoprol eflornithine combination therapy (n = 1) or eflornithine nifurtimox combination therapy (n = 1). Patients were monitored for treatment outcome during 2 years. The detailed description of the clinical outcomes in the cohort is given elsewhere [11]. In brief, out of 242 primary cases, the final outcome was cure in 90 (cure or probable cure) and treatment failure in 118 (relapse, probable relapse, or HAT related death during follow-up). 34 primary cases were excluded from the analyses of post-treatment results since they could not be classified as cured or treatment failure because they were lost to follow-up, died during treatment or died over the following 2 years from non-HAT related causes. Out of the 118 retreatment cases, 85 were cured and 16 experienced a new treatment failure. Seventeen retreatment cases were lost to follow-up, died during treatment or died over the following 2 years from non-HAT related causes and were also excluded from the analyses of post-treatment results.

### CATT test

CATT/*T.b. gambiense* was performed following the titration-method as described by the manufacturers [6] on serum taken before treatment and at 3, 6, 12, 18 and 24 months post-treatment. The end titre (highest dilution giving agglutination) was determined. Patients with end titres ≥1∶4 were considered CATT positive, end titres <1∶4 were considered CATT negative.

### Data analysis

The Chi square test or Fisher exact test (when the number of observations in a cell was <5) was performed for comparison of proportions using a 95% confidence limit. Odds ratios (OR) with binomial 95% confidence intervals (CI) were computed. STATA version 10 was used for data analysis.

## Results

### CATT before treatment

**Figure 1:**
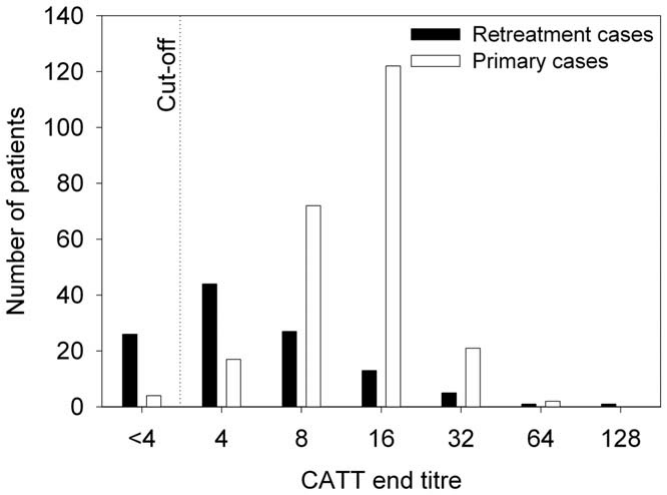
Distribution of CATT end titres in primary and retreatment sleeping sickness cases at inclusion in the study.

The distribution of CATT end titres in primary and retreatment cases at inclusion is presented in figure 1. The median end titre in primary cases was 1∶16 (interquartile range [IQR] 1∶8–1∶16, mean±standard deviation: 14±9), while it was 1∶4 (IQR 1∶4–1∶8, mean±standard deviation: 8±14) in the retreatment cases included in the cohort. Sensitivity of CATT was 98.3% in primary cases (234/238, CI 95.8–99.5%) and 77.8% (91/117, CI 69.2–84.9%) in retreatment cases. The median time between previous and current treatment in retreatment patients was 10 months (IQR 6–16 months, data available for 103/118 cases).

### CATT after treatment

**Figure 2:**
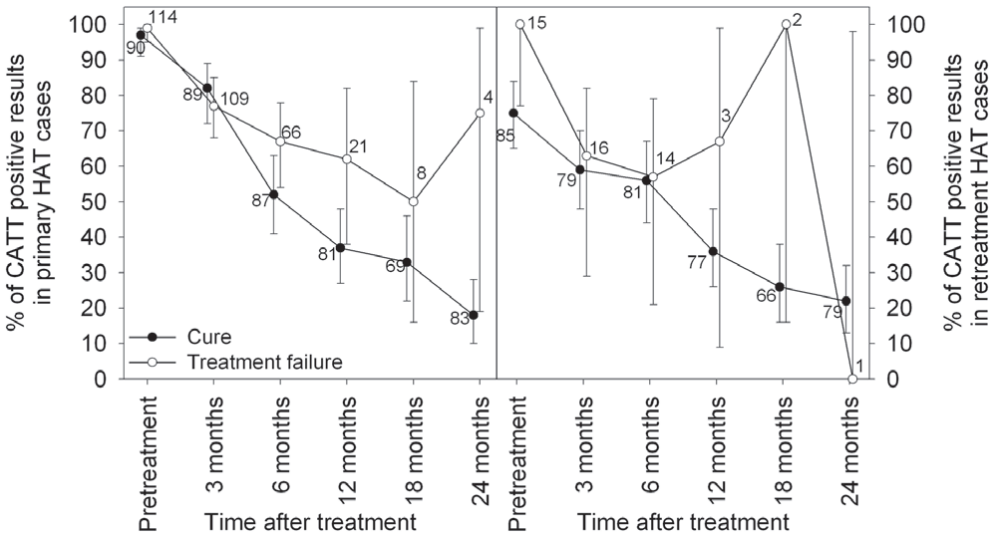
CATT positivity (%) during follow-up in primary HAT (left) and retreatment cases (right) who were cured or experienced treatment failure after current treatment. The number of samples that was tested is indicated, whiskers show 95% confidence intervals.

The CATT results after treatment - in function of cure or treatment failure- are shown in figure 2.

In the 90 cured primary HAT cases, the median end titre decreased to 1∶8 (IQR 1∶4–1∶8) and 1∶4 (IQR <1∶4–1∶8) after 3 and 6 months respectively, and became <1∶4 afterwards. As shown in figure 2, the proportion of CATT positives decreased in the cured group over time to 52% (45/87) and 37% (30/81) at 6 and 12 months and to 18% (15/83) at the final follow-up visit at 24 months (also called test of cure). In the 118 primary cases who experienced treatment failure within the 2 years of follow-up, the median end titre decreased to 1∶8 (IQR 1∶4–1∶8) after 3 months and 1∶4 (<1∶4–1∶8) after 6 and 12 months. The proportion of CATT positives also decreased in function of time to 67% (44/66) and 62% (13/21) at respectively 6 and 12 months after treatment.

No significant relationship between CATT positivity and occurrence of treatment failure (*p*>0.05) could be observed at 3, 6 and 18 months post-treatment. A significantly higher proportion of treatment failures cases tested positive with the CATT compared to the cases that were cured 12 months (Chi square test, *p* = 0.040) and 24 months (Fisher exact test, *p* = 0.027) after treatment. The odds of a treatment failure case being CATT positive are 2.76 (95% CI 1.03–7.4) and 13.6 (95% CI 1.32–140) times greater than the odds of a cured case being CATT positive 12 and 24 months after treatment. In 7/113 primary cases trypanosomes were detected in blood at time of relapse. Two of them relapsed at 3 months with CATT titres 1∶8 and 1∶16 ; three others showed a titre 1∶4 (relapses at 6 and 12 months) and two relapsed at 24 months with titres 1∶8 and 1∶16.

In the 85 retreatment cases who were cured after the current treatment, the median end titre was 1∶4 (IQR <1∶4–1∶8, IQR <1∶4–1∶4) after 3 and 6 months, and became <1∶4 afterwards. The proportion of CATT positives decreased over time to 56% (45/81) and 36% (28/77) at 6 and 12 months and 17% at the 24 months test of cure visit (figure 2). In 16 retreatment cases that experienced a repeated treatment failure after the current treatment, the median end titre was 1∶4 (IQR <1∶4–1∶4) and the proportion of CATT positives 57% (8/14) 6 months post-treatment. No significant relationship between CATT positivity and occurrence of treatment failure (*p*>0.05) could be observed during follow-up. In 3/16 of retreatment cases trypanosomes were detected in blood at time of relapse. Their CATT titres were <1∶4 (relapse at 3 months), and <1∶4 and 1∶16 (relapses at 6 months).

## Discussion

We demonstrate for the first time that CATT sensitivity is low in retreatment cases, and that CATT titres decrease after treatment both in patients who experience treatment failure as well as in cured patients.

Before treatment, the CATT sensitivity in primary cases falls within the sensitivities previously reported for CATT in the Democratic Republic of the Congo, and for HAT in general [7],[10]. The low sensitivity of 78% observed in retreatment cases is explained by the decrease in CATT titre after a previous treatment, and largely corresponds to the proportion of CATT positives observed 6 and 12 months post-treatment within the groups of treatment failures. The observed end titers are relatively low, being in respectively 39% and 83% of the primary and retreatment cases below 1∶16. Treating serological cases based on a CATT end titer ≥1∶16, without parasitological evidence, might miss some HAT cases.

Although it has been shown that trypanosome specific antibody concentrations in blood of cured patients may persist up to 2 years or longer after treatment [3],[9],[10],[12], reports about the concentrations of specific antibodies in serum of sleeping sickness patients who experience treatment failure are rare. In 22 relapsing patients, Frézil et al. [12] describe that the immunofluorescence test remains positive in the majority of relapsing cases, but doubtful/negative in only 1 case. In a small cohort of 32 relapse cases, Miézan et al. [3] describe a decreasing antibody concentration and a CATT positivity rate of 94% at the moment relapse is diagnosed.

A negative CATT after unsuccessful treatment might be explained by trypanosomes that are cleared from peripheral tissues, such as lymph and blood, but that survive in the brain and thus do not trigger specific antibody production in the blood.

Our study has a number of limitations. As a consequence of the diagnostic procedure used by the HAT control program to detect HAT, the observed sensitivity of CATT of 98% in our group of primary cases might be higher than in other patient cohorts. Indeed, the patients in our cohort were identified as follows: CATT on whole blood, alongside cervical lymph node palpation, was used as a screening test and only those persons with a CATT positive result on whole blood, or having enlarged cervical lymph nodes underwent parasitological examinations for case confirmation. Although part of the false negatives in CATT will be found by cervical lymph node palpation, the true sensitivity of CATT in the primary cases might be lower than 98%. The number of treatment failures detected after ≥12 months is low, which prevents us from making reliable estimates of a further de- or increase in CATT titres after that time point, nor of the proportion of CATT positives. Moreover, follow-up examinations in this cohort- as in routine clinical care- were focused on cerebrospinal fluid examination and the need for blood examinations may have been given less importance by the nursing staff. As a consequence the cohort does not allow us to check if relapsing patients with trypanosomes in the blood had higher CATT titres than those without, since in the majority, relapse was confirmed by finding of trypanosomes in the CSF and no further blood examinations were performed. Finally, the majority of primary cases in this study were treated with the trypanolytic drug melarsoprol in an area of high treatment failure rates. Although a similar trend was observed in retreatment cases, who were treated differently, we cannot exclude that results could differ in primary HAT patients treated with other drugs.

Our findings have 2 practical implications. First, the considerable proportion of CATT negative results in cases experiencing treatment failure, which increases over time, implies that a post-treatment CATT negative result does not necessarily indicate cure. Knowing, moreover that a post-treatment CATT positive result does not indicate treatment failure, makes us conclude that CATT is unreliable for monitoring treatment outcome. Secondly, screening programs for HAT should take into consideration that a careful history about past HAT episodes is paramount, as the sensitivity of CATT in relapse cases is not optimal. Cases experiencing treatment failure are more likely to be false negative in CATT than new cases and, as a consequence, might be missed (i.e. not offered parasitological investigations) if they show no clinical signs. These data might cast some doubt on the performance of CATT as a screening test in the detection process, given the fact that some relapse cases appear to be negative in the CATT. Molecular -or other- diagnostics might eventually be taken up in an improved algorithm for diagnosis or follow-up but further investigation of these tests is necessary.
